# Association of seeking ophthalmic assessment in children with parental history of refractive errors

**DOI:** 10.12669/pjms.35.3.1010

**Published:** 2019

**Authors:** Muhammad Zahid Latif, Muhammad Athar Khan, Saira Afzal, Syed Amir Gilani

**Affiliations:** 1 *Dr. Muhammad Zahid Latif, MBBS, MPH, MME, PhD (RS) Department of Community Medicine & Medical Education, Azra Naheed Medical College, The Superior University Lahore, Pakistan*; 2 *Dr. Muhammad Athar Khan, MPH (USA), PhD (USA), Institute of Public Health, The University of Lahore, Lahore, Pakistan*; 3 *Dr. Saira Afzal MBBS, MPhil, MCPS, FCPS, PhD, Dean Faculty of Community Medicine & Public Health, King Edward Medical University, Lahore, Pakistan*; 4 *Dr. Syed Amir Gilani, MBBS, DMRD, PhD, Dean Faculty of Allied Health Sciences, The University of Lahore, Lahore, Pakistan*

**Keywords:** Astigmatism, Myopia, Ophthalmic examination, Refractive Errors, School children

## Abstract

**Objective:**

To find out the association of seeking ophthalmic assessment in children with parental history of refractive errors.

**Methods:**

After the approval of ethical review board, an analytical cross-sectional study was conducted in eight high schools of public and private sector at Lahore during the period of seven months from August 2017 to March 2018. Multistage random sampling technique was opted and 2000 study subjects were recruited including 50% boys and 50% girls. Informed consent was obtained and data was collected on a structured questionnaire. The data was organized, entered in version 23 of IBM SPSS and analyzed by the use of statistical tools.

**Results:**

Age of the respondents ranged between nine to 18 years with a mean of 13.40 ± 1.82 SD.

Parental history of wearing spectacles was present in 21.3% of the fathers and 28.6% of the mothers. Moreover, 72.4% of the participants never visited eye care professional. Among private schools, an association was found between the visit of boys to eye care professional and maternal positive history of wearing spectacles (p-value 0.019). A significant association was found between the positive paternal history of wearing spectacles and visit of the female strudy subjects to an eye care professional (p-value 0.001). In public schools, there was an association between visit of children to eye care services and positive history of mothers about the use of spectacles (p-value 0.018).

**Conclusions:**

This study concludes that positive maternal history of wearing spectacles is associated with the ophthalmic examination of children in both public and private school.

## INTRODUCTION

Refractive errors are a significant challenge for public health and considered as the commonest ophthalmic problem.^1^ Globally, around 259 million people are visually impaired including 98 million having refractive errors.^2^ Refractive errors are the most common reason for the visit of an individual to eye care professional and 43% of the total visual impairment is due to refractive errors.^3^ Children are the most common victim and these errors are directly linked with poor quality of life, nutritional deficiencies, educational achievements and economic loss.^4^ However, the solution for this problem is a low-cost health care intervention and 80% of the causes of visual impairment can be prevented or treated to avoid blindness.^5^ The available literature identifies that a significant number of Pakistani school children are suffering from the refractive errors. A relevant study concluded that every 5^th^ school going child is having a visual disability in the form of refractive errors in Pakistan.^6^

Different reasons for the non-correction of refractive errors have been concluded in literature. It includes poor knowledge of the issue, lack of services, affordability and accessibility. The economically viable communities with undetected or uncorrected refractive errors in children have also been reported.^7^ Bonnie Keaton found in his study that around 80% of children in preschool age never receive an ophthalmic examination. Major barriers concluded through this research include inconvenience, unawareness and attitude of parents for eye care.^8^ The study described the attitude of parents as the leading problem to seek eye care services for the children. This means affordability, accessibility and approach to the ophthalmic facilities are relevant issues but changing the affective domain of parents for ophthalmic examination of children is still a problem. Another research revealed that the support of parents is a massive issue for visual screening. This study concluded that after around eighteen months of referral, the parents get the child examined by an eye specialist.^9^ On the other side, this is based on evidence that positive family history of refractive errors enables parents to pursue for the ophthalmic examination and care of the children.^10^ It is also important to mention that the genetic contribution is a major factor regarding the etiology of refractive errors. So, parental attitude towards ophthalmic care should be considered as a key component to improve the visual screening drives in children.

Apart from the financial resources, accessibility, relevant awareness, preventive practices adopted by parents, visual aids and provision of nutritional supplements are fundamental steps for prevention of blindness in the children. It is worth mentioning that parents work hard and remain committed to uplift and educate their children. However, ignoring the vital component of visual screening is an important question mark. The relevant literature about parental history of spectacles and ophthalmic examination of their children especially in Pakistani context is scarce. In view of the above-mentioned scenario, this study was designed and conducted to find out the association between parental history of wearing spectacles and ophthalmic assessment of the children.

## METHODS

An analytical cross-sectional study was conducted in eight high schools of public and private sector at Lahore Pakistan during the period of seven months from August 2017 to March 2018. The study was ethically approved by the Institutional Ethical Review Board (IERB) of The University of Lahore. Multistage random sampling technique was used.^11^ Out of the five Tehsils, one was randomly selected, a list of the number of union councils (UC) in the selected Tehsil was obtained and one UC in urban setting and one UC from rural setting were randomly selected. Later, a list of both public and private high schools in both the UCs was obtained and four public and four private schools from both settings were included in the study. Fifty students of class 6 to class 10 were included whereas the students of other classes were excluded from the study. The sample size was calculated by the Open Epi Tool Kit and following formula was used^12^;

α Level of significance 95.00%

P1 Expected Proportion of children with refractive error 20.00%^6^

d Expected error 6.00%


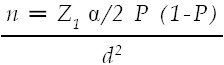


n Sample size in one group 171

**Fig.1:**
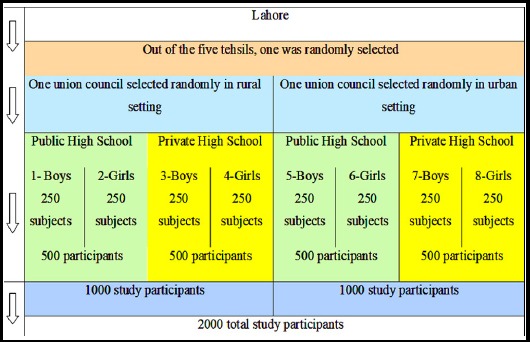
Flow diagram showing the selection of study subjects.

The estimated sample size for each group was 171 study subjects. However, to increase the strength, accuracy and precision, 250 subjects were included in each group leading to a total of 2000 study subjects. The distribution of the study subjects is presented in Fig.1.

Screening of refractive errors, according to the defined protocols with required instruments (Snellen charts, refraction boxes with trial frames, autorefractometer, retinoscope, ophthalmoscope and hand-held slit lamp etc.) and logistics by a team of optometrists supervised by an ophthalmologist was managed in the schools.^13^ Visual Acuity (VA) of the study subjects was evaluated with the Standard Snellen Chart placed at a distance of six meters. Students with VA less than 6/9 in the better eye or both eyes were tested for the presence of refractive error by a pinhole. The improvement of the vision with pinhole examination was followed by auto and subjective refraction. It was also cross-checked by retinoscopy. Data was collected on a self-structured questionnaire which consisted of different parts, including basic profile of the participants, relevant family history, ever visit to an eye care professional for visual assessment, ophthalmic examination, VA, best correction, diagnosis and type of refraction. The questionnaire was discussed with experts in ophthalmology to ensure its validity and also pretested for reliability. Informed consent was obtained from the study subjects and permission of parents was also ensured through the school administrations.

The collected data was organized and entered in version 23 of the software Statistical Package for Social Sciences (SPSS). The data was analyzed by using the Chi-square test and a p-value of ≤0.05 was considered as significant.

## RESULTS

There were 2000 subjects with a mean age of 13.40 ± 1.82 SD (range: 9-18 years). The overall prevalence of refractive errors calculated in this study was 20.6%. Myopia was the leading type 52.2% followed by Astigmatism 33% and Hypermetropia 14.8% respectively. The history of wearing spectacles among parents of the study subjects was studied and found that it was positive in 21.3% of the fathers and 28.6% of the mothers. Similarly, any visit of the study subjects to an eye care professional for ophthalmic examination or vision testing was investigated. It was found that 72.4% of the study participants never visited ophthalmic services for clinical examination.

The parental history of the use of spectacles and ever visit of the study subjects to eye care professional for ophthalmic assessment among the private schools were compared and results are presented in Table-I. The findings represent that there was no association between the studied variables. However, a significant association was found between the positive maternal history of the use of spectacles and ever visit of the male study subjects to an eye care professional (p-value 0.019). These male study participants were not having refractive errors (Table-I). A significant association was also found between the visit of the female study subjects to an eye care professional and positive paternal history about the use of spectacles (p-value 0.001). Interestingly, refractive errors were not prevelant in these study participants (Table-I)

**Table-I:** Comparison of Private school students according to the parental history of wearing spectacles and student’s visit to eye care professional.

Gender	Refractive Error	Ever visited eye care professional	Mother’s History of glasses			Father’s history of glasses		

			Yes (%)	No (%)	P-value	Yes (%)	No (%)	P-value
Male	Yes	Yes	10 (24.4)	31 (75.6)	0.188	11 (26.8)	30 (73.2)	0.722
		No	2 (9.1)	20 (90.9)		5 (22.7)	17 (77.3)	
		Total	12 (19.0)	51 (81.0)		16 (25.4)	47 (74.6)	
	No	Yes	16 (34.0)	31 (66.0)	0.019*	9 (19.1)	38 (80.9)	0.245
		No	72 (18.5)	318 (81.5)		47 (12.1)	343 (87.9)	
		Total	88 (20.1)	349 (79.9)		56 (12.8)	381 (87.2)	
Female	Yes	Yes	24 (35.3)	44 (64.7)	0.666	20 (29.4)	48 (70.6)	0.256
		No	11 (29.7)	26 (70.3)		7 (18.9)	30 (81.1)	
		Total	35 (33.3)	70 (66.7)		27 (25.7)	78 (74.3)	
	No	Yes	16 (29.6)	38 (70.4)	0.101	19 (35.2)	35 (64.8)	0.001*
		No	65 (19.1)	276 (80.9)		53 (15.5)	288 (84.5)	
		Total	81 (20.5)	314 (79.5)		72 (18.2)	323 (81.8)	

* p-value significant at ≤0.05, using Chi-square test

The results regarding comparison of the study subjects from public-schools regarding parental history of wearing spectacles and student’s visit to eye care professional are presented in Table-II. There was no association but a positive maternal history of the use of spectacles was significantly associated with the ever visit of the male study subjects having refractive errors to an eye care professional (p-value 0.018).

**Table-II:** Comparison of Public-school students according to History of wearing spectacles in parents and student’s visit to eye care professional.

Gender	Refractive error	Visited eye care professional	Mother’s History of glasses			Father’s history of glasses		

			Yes (%)	No (%)	P-value	Yes (%)	No (%)	P-value
Male	Yes	Yes	34 (50.7)	33 (49.3)	0.018*	18 (26.90)	49 (73.10)	0.362
		No	12 (27.3)	32 (72.7)		8 (18.20)	36 (81.80)	
		Total	46 (41.4)	65 (58.6)		26 (23.40)	85 (76.60)	
	No	Yes	33 (34.4)	63 (65.6)	0.707	28 (29.20)	68 (70.80)	0.095
		No	94 (32.1)	199 (67.9)		61 (20.80)	232 (79.20)	
		Total	127 (32.6)	262 (67.4)		89 (22.90)	300 (77.10)	
Female	Yes	Yes	40 (44.9)	49 (55.1)	0.855	27 (30.30)	62 (69.70)	0.549
		No	19 (43.2)	25 (56.8)		11 (25.00)	33 (75.00)	
		Total	59 (44.4)	74 (55.6)		38 (28.60)	95 (71.40)	
	No	Yes	38 (42.2)	52 (57.8)	0.073	24 (26.70)	66 (73.30)	0.690
		No	87 (31.4)	190 (68.6)		80 (28.90)	197 (71.10)	
		Total	125 (34.1)	242 (65.9)		104 (28.30)	263 (71.70)	

* p-value significant at ≤0.05, using Chi-square test

## DISCUSSION

Refractive error is a major public health challenge for the global communities especially in developing countries.^14^ The effected part of the population belongs to various age groups but a significant number of the school children become the victim leading to serious individual, national and international consequences.^15^ According to the report of American Optometrist Association, approximately 25% of school age children have problems related to vision.^9^ The current study was conducted in the high schools of Lahore which is the second largest city of Pakistan, provincial head quarter and also considered as the educational hub of the country. The findings of the present study conclude prevalence of refractive errors as 20.6% of the total study subjects. These results are contradictory to the finding of a study from Iraq concluding a prevalence of 33%.^16^ But the results about types of refractive errors are consistent with the findings of this study as Myopia has been concluded as the major type in both the studies.^16^ However, it is worth mentioning that the above-mentioned cross-sectional research was conducted among the students of a medical college. Similarly, the findings regarding prevalence of refractive errors (20.6%) are contradictory to the results of another relevant study conducted in school children at Ankara, Turkey concluding a prevalence of 10.8%.^17^ The findings of the present study are close to the results of another similar research conducted among the students of class 6^th^ to 10^th^ at district Kancheepuram of India concluding the prevalence of refractive errors as 23.8%.^18^ The results regarding prevalence of refractive errors are similar to the finding of a study conducted among the primary school students of Muzaffarabad concluding a prevalence of 19.6%.^19^ However the findings are quite different from the results of another school based study conducted at Public sector schools of Bangalore India in which the found prevalence of refractive errors was 10%.^20^ Similarly, the results about the ever visit to an eye care professional for ophthalmic examination are contrary to the finding of above-mentioned study. The current study concludes that 27.8% of the study subjects ever visited any eye care professional whereas the study conducted in Bangalore found that 10.9% of the students were ever checked for ophthalmic examination.^20^ A study by Parrey and colleagues from Saudi Arabia has reported the prevalence of RE to be 45.8% which is much higher that the prevalence of RE in our study 20.6%. The commonest type of RE in their study was myopia in 24.4% followed by hyperopia in 11.9% and simple astigmatism in 9.5% cases.^21^ However, this research was conducted among adults aged 16 to 39 years.

The focus of this study was to find the association between parental history of spectacles use and any visit of their children to eye care professionals to seek clinical care. It was supposed that the parents with the history of refractive errors and use of required spectacles do have the better access to eye services in terms of knowledge and care. Research studies reveal that the cooperation of parents for ophthalmic examination and subsequent management of the issue is still a major barrier for the school vision programs.^22^ Diverse studies reveal that refraction is associated with genetic factors and positive family history empowers parents to pursue for the care of children.^10^,^23^

The results of present study, among the private schools about parental history of wearing spectacles and the visit of child to eye care professional conclude that an association was found between the visit of boys to eye care professional and positive maternal history regarding the use of spectacles (p-value 0.019). It is important to mention that these boys were not having the refractive errors as diagnosed during this study. On the other side, an association was found between the visit of girls to an eye care professional and positive paternal history about the use of spectacles (p-value 0.001). The relevant findings among the public-school participants conclude an association between positive maternal history of spectacles and the visit of boys to ophthalmic services for clinical examination (p-value 0.018). It is worth mentioning that these study subjects were having refractive errors. However, there was no association between parental history of spectacles use and ever visit of the child to eye care professional. The findings are quite interesting reflecting the gender based societal practices. Association of maternal history of spectacles use with clinical examination of boys and paternal history with ophthalmic assessment of girls represent the local cultural values. The relevant literature concludes that male gender-oriented spending are common in Asian countries like China, India and Pakistan especially for education and health.^24^ However, no differential care seeking for boys and girls was found in a study about the gender-based determination of household decision for health care conducted in Thatta, Pakistan.^25^ This is an initial study about an important question resulting in significant finding. The limitation of this research is the study of association of parental history with the visit of child to eye care professional only and other relevant factors such as parental socio economic and educational status have not been investigated. However, the study provides a baseline for further research in this area.

## CONCLUSION

The study concludes that the gender oriented cultural preferences influence the eye care seeking behavior of the parents for their children. It describes the importance of Women’s role as positive maternal history of wearing spectacles is associated with seeking professional care for the children in both public and private school. It reflects that mothers are more concerned for clinical assessment of the children.

### Authors’ Contribution

**MZL** conceived, designed, collected data, analysis and initial write up.

**MAK & SA** statistical analysis, interpretation, edited the manuscript & reviewed.

**SAG** reviewed and finalized the article.
